# Ancient papillomavirus-host co-speciation in Felidae

**DOI:** 10.1186/gb-2007-8-4-r57

**Published:** 2007-04-12

**Authors:** Annabel Rector, Philippe Lemey, Ruth Tachezy, Sara Mostmans, Shin-Je Ghim, Koenraad Van Doorslaer, Melody Roelke, Mitchell Bush, Richard J Montali, Janis Joslin, Robert D Burk, Alfred B Jenson, John P Sundberg, Beth Shapiro, Marc Van Ranst

**Affiliations:** 1 Laboratory of Clinical & Epidemiological Virology, Rega Institute for Medical Research, University of Leuven, Minderbroedersstraat, B3000 Leuven, Belgium; 2 Department of Zoology, University of Oxford, South Parks Road, Oxford OX1 3PS, UK; 3 Department of Experimental Virology, Institute of Hematology and Blood Transfusion, U Nemocnice, 128 22 Prague, Czech Republic; 4 The Brown Cancer Center, University of Louisville, South Jackson Street, Louisville, KY 40202, USA; 5 Department of Epidemiology and Social Medicine, Comprehensive Cancer Center, Albert Einstein College of Medicine, Morris Park Avenue, Bronx, NY 10461, USA; 6 Basic Research Program-SAIC Frederick-National Cancer Institute, Building 560, Frederick, MD 21702-1201, USA; 7 National Zoological Park, Smithsonian Conservation and Research Center, Remount Road, Front Royal, VA 22630, USA; 8 East Wakefield Drive, Alexandria, Virginia 22307, USA; 9 Phoenix Zoo, Galvin Parkway, Phoenix, AZ 85008, USA; 10 The Jackson Laboratory, Main Street, Bar Harbor, MA 04609-1500, USA

## Abstract

**Background:**

Estimating evolutionary rates for slowly evolving viruses such as papillomaviruses (PVs) is not possible using fossil calibrations directly or sequences sampled over a time-scale of decades. An ability to correlate their divergence with a host species, however, can provide a means to estimate evolutionary rates for these viruses accurately. To determine whether such an approach is feasible, we sequenced complete feline PV genomes, previously available only for the domestic cat (*Felis domesticus*, FdPV1), from four additional, globally distributed feline species: *Lynx rufus *PV type 1, *Puma concolor *PV type 1, *Panthera leo persica *PV type 1, and *Uncia uncia *PV type 1.

**Results:**

The feline PVs all belong to the Lambdapapillomavirus genus, and contain an unusual second noncoding region between the early and late protein region, which is only present in members of this genus. Our maximum likelihood and Bayesian phylogenetic analyses demonstrate that the evolutionary relationships between feline PVs perfectly mirror those of their feline hosts, despite a complex and dynamic phylogeographic history. By applying host species divergence times, we provide the first precise estimates for the rate of evolution for each PV gene, with an overall evolutionary rate of 1.95 × 10^-8 ^(95% confidence interval 1.32 × 10^-8 ^to 2.47 × 10^-8^) nucleotide substitutions per site per year for the viral coding genome.

**Conclusion:**

Our work provides evidence for long-term virus-host co-speciation of feline PVs, indicating that viral diversity in slowly evolving viruses can be used to investigate host species evolution. These findings, however, should not be extrapolated to other viral lineages without prior confirmation of virus-host co-divergence.

## Abstract 2

The evolutionary rate of feline papillomaviruses is inferred from the phylogenetic analysis of their hosts, providing evidence for long-term virus-host co-speciation

## Background

Understanding demographic processes in populations has been a fundamental challenge in evolutionary biology and population genetics. Inference is often limited by the slow nature of the accumulation of genetic diversity, particularly in vertebrate populations, often resulting in a lack of statistical power [1]. One way to circumvent this problem is to use changes accumulating in rapidly evolving genetic markers, such as associated pathogens, to infer the evolutionary history of the host. This approach was recently used to investigate demographic and phylogeographic patterns in cougar populations (*Puma concolor*), for which host microsatellite data revealed only low genetic differentiation [2]. In the same way, it may be possible to use more slowly evolving viruses to reconstruct evolutionary relationships between host species.

In fast evolving pathogens, genetic sequences usually accumulate sufficient substitutions over relatively limited periods of time, which allows their evolutionary rates to be estimated reliably. For such 'measurably evolving populations', the molecular clock can hence be calibrated using temporal information in serially samples sequences [3]. However, this is not the case for slowly evolving viruses such as papillomaviruses (PVs), for which viral sequences sampled decades apart are almost identical and should be considered as contemporaneous, given the time frame of PV evolution. This was demonstrated by the finding that two isolates of bovine BPV1, collected from remote cattle populations (from Sweden and the USA) and approximately 30 years apart, had nearly identical sequences; only five differences were found when comparing 4,807 nucleotides that covered the entire late region and part of the early region of the genomes [4]. Furthermore, the lack of fossil calibration has made it practically impossible to determine longer term rates of evolution for these slowly evolving viruses. If viruses co-evolve with hosts, however, it may be possible to use host fossil calibration points to calibrate the viral phylogeny, providing a mechanism to address interactions between populations or species over longer evolutionary time-frames. The slowly evolving and species-specific PVs provide excellent candidates in which to test the feasibility of this approach.

The *Papillomaviridae *are a large family of small non-enveloped, double-stranded DNA viruses. They can cause benign and malignant proliferations of the stratified squamous epithelium of the skin and mucosa in a wide variety of vertebrate species. PVs are highly species specific, or at least they are restricted to infection of closely related animal species, and it is likely that most mammal and bird species carry their own set of PV types.

PV-containing lesions were described in six feline species: the domestic cat (*Felis domesticus*), bobcat (*Lynx rufus*), Florida panther (*Puma concolor coryi*, previously named *Felis concolor coryi*), Asian lion (*Panthera leo persica*), snow leopard (*Uncia uncia*, previously named *Panthera uncia*), and the clouded leopard (*Neofelis nebulosa*) [5,6]. To date, the *Felis domesticus *PV type 1 (FdPV1) is the only feline PV that was isolated and completely genomically characterized. This FdPV1 was found to be closely related to the domestic dog (canine) oral PV (COPV), and both viruses possess a unique noncoding intervening sequence between the end of the early and the beginning of the late protein coding region of their genome [7,8]. FdPV1 and COPV are classified in two different species of the genus Lambdapapillomavirus (λ-PV) [9]. Based on the close relationship between FdPV1 and COPV, and between their Canidae and Felidae hosts, we suggested that the most recent common ancestor of these viruses was present in a common ancestor of cats and dogs, and was passed on to the Canidae and Felidae descendent lineages, which subsequently started to diverge [8].

This report describes the complete sequencing and evolutionary analysis of four novel felid PVs: *Lynx rufus *PV type 1 (LrPV1), *Puma concolor *PV type 1 (PcPV1), *Panthera leo persica *PV type 1 (PlpPV1), and *Uncia uncia *PV type 1 (UuPV1). Our analyses demonstrate that the evolutionary history of the feline PVs is closely linked to that of their feline hosts, indicating that the host phylogeny can be used to calibrate the viral evolutionary clock, or conversely that viral diversity in slowly evolving viruses can provide a means for unraveling ancient host evolutionary processes.

## Results

### Genomic sequences of LrPV1, PcPV1, PlpPV1, and UuPV1

The PV sequences reported in this paper were isolated from an oral papillomatous lesion on the tongue of a bobcat, a lesion under the tongue of a Florida panther, a papillomatous lesion on the ventral surface of the tongue of an Asian lion, and a lesion on the lower lip of a snow leopard. From these lesions, the complete genomes of four novel PVs were cloned and sequenced: LrPV1 (8,233 base pairs [bp]; GenBank: AY904722), PcPV1 (8,321 bp; GenBank: AY904723), PlpPV1 (8,103 bp; GenBank: AY904724), and UuPV1 (8,078 bp; GenBank: DQ180494).

All characterized PVs have their open reading frames (ORFs) on one strand of their circular double-stranded DNA genome, and the ORFs on this coding strand are classified as either early (E) or late (L), based on the location in the genome. The LrPV1, PcPV1, PlpPV1, and UuPV1 sequences contain the seven classical PV major ORFs, encoding five early proteins (E1, E2, E4, E6, and E7) and two late capsid proteins (L1 and L2). The exact locations of the ORFs and the size of the predicted proteins, with a comparison with the corresponding ORF data from FdPV1, are summarized in Additional data file 1. The position of the first nucleotide of the genomes was fixed corresponding to the start of the first major ORF in the early region (E6).

In the early region of the feline PV genomes, canonical E6 and E7 ORFs are present, which encode the major papillomaviral transforming proteins. The predicted E6 proteins of LrPV1, PcPV1, PlpPV1, and UuPV1 contain two conserved zinc-binding domains, separated by 35 amino acids. The first domain exhibits the classical C-X-X-C-X_29_-C-X-X-C motif, whereas the second motif is modified, containing only 28 amino acids (X_28_) between the two instances of C-X-X-C. An amino acid alignment of the E6 of LrPV1, PcPV1, PlpPV1, UuPV1, and 43 PV type species that contain an E6 revealed that only the canine COPV, the feline FdPV1, and the raccoon (*Procyon lotor*) PlPV1 (all members of the λ-PV genus) have an identical X_28 _modified motif. The only other nonclassical motifs were identified in the cottontail rabbit CRPV (X_33_), the rabbit oral ROPV (X_34_), and the equine (*Equus caballus*) EcPV1 (X_30_). The E7 contains one zinc-binding domain, also with the modified X_28 _motif in LrPV1, PcPV1, PlpPV1, COPV, PlPV1, and FdPV1. The UuPV1 contains a different X_26 _modified zinc-binding motif. The E7 of LrPV1, PcPV1, PlpPV1, and UuPV1 also contains the conserved retinoblastoma tumor suppressor binding domain with the consensus sequence DLRCYEQMP(D/G)EEE.

The E1 ORF encodes the largest PV protein (606 amino acids in PcPV1, PlpPV1, and UuPV1, and 608 amino acids in LrPV1), and contains the conserved ATP-binding site of the ATP-dependent helicase (GPPNTGKS) in the carboxyl-terminal part. Papillomaviral E1 proteins have DNA-dependent ATPase and DNA helicase activities, and are essential for both the initiation and elongation of viral DNA synthesis. The E2 gene product is a DNA binding protein that functions as an important regulator of viral transcription and replication, and a conserved leucine zipper domain (L-X_6_-L-X_6_-L-X_6_-L) is present in the carboxyl-terminal part of E2. The E4 ORF is completely contained within the E2 gene. As is the case in most PVs, the E4 does not possess a start codon. In the human PVs (HPVs) that have been studied, E4 is primarily expressed from a viral transcript formed by splicing a few codons from the beginning of E1 to E4. Although the function of the E4 protein has not been completely clarified, current data suggest that it may assist in viral release from the infected cells through association with the cytoskeleton [10]. In the late region, the major (L1) and minor (L2) capsid protein genes are present. Both L1 and L2 contain a series of highly basic amino acid residues (Arg and Lys) at their carboxyl-terminus, probably functioning as a nuclear localization signal.

**Figure 1:**
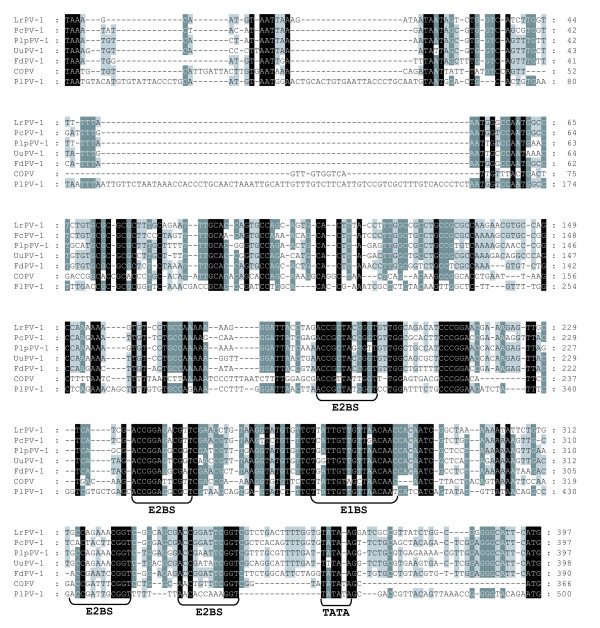
URR alignment of the λ-PVs. Nucleotide sequence alignment of the upstream regulatory region (URR) or noncoding region (NCR1), from the stop codon of L1 to the start codon of E6, including the feline papillomaviruses (PVs) *Felis domesticus *PV type 1 (FdPV1), *Lynx rufus *PV type 1 (LrPV1), *Puma concolor *PV type 1 (PcPV1), *Panthera leo persica *PV type 1 (PlpPV1), and *Uncia uncia *PV type 1 (UuPV1), and the nonfeline carnivore PVs canine oral PV (COPV) and *Procyon lotor *PV type 1 (PlPV1), all of which belong to the genus λ-PV. Nucleotides are shaded according to the degree of conservation (black = 100% conserved; dark gray = 80% to 99% conserved; light gray = 60% to 79% conserved; no shade = <60% conserved). The conserved and putative E2 binding sites (E2BSs) and the E1 binding site (E1BS) are indicated. In LrPV-1, an E1BS is located at nucleotides 8,151-8,167, flanked by two conserved E2BSs at nucleotides 8,068-8,079 and 8,215-8,226. Two additional modified putative E2BSs were identified at nucleotides 8,117-8,128 (ACC-N6-GTT) and 8,195-8,206 (GCC-N6-GGT). The E1BS in PcPV-1 is located at nucleotides 8,226-8,242, surrounded by conserved E2BSs at nucleotides 8,143-8,154 and 8,289-8,300, and putative modified E2BSs at nucleotides 8,193-8,204 (ACC-N6-GTT) and 8,269-8,280 (CAC-N6-GGT). In PlpPV-1 we identified an E1BS at nucleotide 8,091-4, two conserved E2BSs at nucleotides 8,057-8,068 and 52-63, and modified putative E2BS motifs at nucleotides 8,007-8,018 (ACC-N6-GTT; a position where all other sequences in the alignment exhibited a conserved E2BS) and at nucleotides 32-43 (GCC-N6-GGT). The E1BS in UuPV-1 is located at nucleotides 7,983-7,999, with three conserved E2BSs at nucleotides 7,899-7,910, 7,949-7,960 and 8,047-8,058, and a putative modified E2BS (GCC-N6-GGT) at nucleotides 8,027-8,038. The TATA box of the E6 promotor in LrPV-1, PcPV-1, PlpPV-1, FdPV-1, COPV, and PlPV-1, and a possible degenerate TATA box (TTTAA) in UuPV-1 is indicated.

The classic upstream regulatory region (URR) or noncoding region (NCR1) between the stop codon of L1 and the start codon of E6 contains only 391 nucleotides in LrPV1 (nucleotides 7,885-42), PcPV1 (nucleotides 7,961-30), and PlpPV1 (nucleotides 7,827-114), and 392 nucleotides in UuPV1 (nucleotides 7,717-30). This is similar to FdPV1, in which the URR counts 384 nucleotides [8]. To activate the PV origin of replication, an E1/E2 complex must bind to the URR, which usually contains an E1-recognition site flanked by two E2-binding sites (E2BSs). A nucleotide alignment of the URR of LrPV1, PcPV1, PlpPV1, and UuPV1 with the URR of FdPV1, COPV, and PlPV1 allowed us to locate an E1 binding site (E1BS), and several conserved E2BSs with the consensus sequence ACC-N_6_-GGT. We also found a number of modified E2BSs that, because of their close similarity to homologous conserved E2BSs in the other sequences and their location relative to the E1BS, could be of functional importance. The positions of the conserved and putative E2BSs, the E1BS, and the TATA box of the E6 promotor are indicated in Figure 1.

Apart from the classical URR (NCR1) between the end of L1 and the start of E6, an additional NCR2 between the early and late protein region is present in the genomes of LrPV1 (position 3,641-4,843, 1,203 nucleotides), PcPV1 (3,614-4,898, 1,285 nucleotides), PlpPV1 (3,710-4,769, 1,060 nucleotides), and UuPV1 (3,623-4,661, 1,039 nucleotides). This NCR2 is absent in all other characterized PVs, with the exception of COPV, FdPV1, and PlPV1 [7,8,11]. Therefore, the presence of an NCR2 is a unique feature of the members of the genus λ-PV. No recognizable E1BS, E2BS, or other regulatory and promoter element could be identified in the NCR2.

### Sequence similarity to other papillomaviruses

The mutual sequence similarities of LrPV1, PcPV1, PlpPV1, and UuPV1, and their similarities to FdPV1, COPV, the prototype benign cutaneous HPV type 1, the epidermodysplasia verruciformis associated HPV5, the mucosal high-risk HPV16, and the bovine fibropapillomavirus BPV1 were investigated by pair-wise nucleotide and amino acid sequence alignments of the different ORFs and their proteins (percentages similarity are summarized in Additional data file 2). For all ORFs, LrPV1, PcPV1, PlpPV1, and UuPV1 exhibited the greatest similarity to each other and to the previously characterized FdPV1. The mutual similarity was comparable among all feline PVs and was markedly greater than the similarity to COPV, which was still greater than the similarity to the human cutaneous and mucosal HPV1, HPV5, and HPV16, and to the bovine BPV1.

According to the current PV taxonomic criteria, PV types that share between 71% and 89% nucleotide identity within the complete L1 ORF belong to the same species, and different species within a PV genus share 60% to 70% nucleotide identity in L1 [9]. Based on these criteria, LrPV1, PcPV1, PlpPV1, and UuPV1 can be classified in the same species as FdPV1 (species 2 of the genus λ-PV), with 73% to 85% nucleotide identity in L1 among each other and with FdPV1. All feline PVs belong to the same genus as COPV (species 1 of the genus λ-PV), which is confirmed by the percentage nucleotide identity in L1 (67% to 69%).

### Phylogenetic analysis

**Figure 2:**
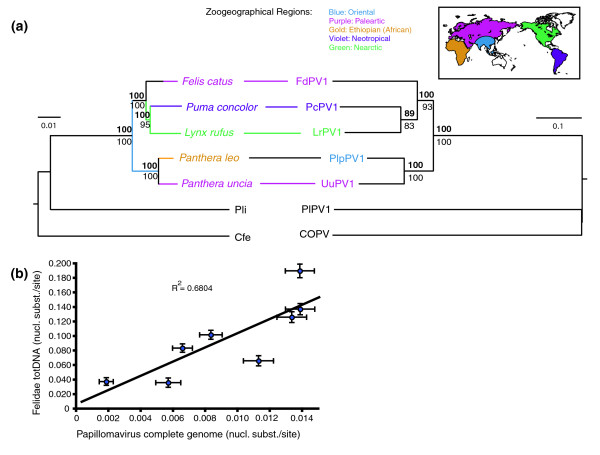
Phylogenetic relations among felid species and their papillomaviruses. **(a) **Co-evolutionary relationships revealed by the feline multigene (left) and papillomavirus (PV) complete coding genome (right) phylogenetic trees. Identical tree topologies were reconstructed by using both maximum likelihood (ML) and Bayesian methods for all data sequences from Johnson and coworkers [12] and for the amino acid translation of the concatenated PV genes. Bayesian posterior support values (bold) and ML bipartition values for relationships are shown on the branches. The scale bars indicate the genetic distance (nucleotide substitutions per site). Scientific names and branches in the host phylogenetic tree are color coded to depict recent and historic zoogeographic regions shown on the map (Oriental, Palearctic, Ethiopian, Neotropical, and Nearctic), as inferred by Johnson and coworkers [12] from current distributions, fossil records, and phylogenetic analysis. Branches in black reflect either less certain historical interpretations or geographic distributions beyond one. For the viral phylogenetic tree, the same color code is used for the abbreviated virus name, depicting the geographical region from which the respective virus was isolated. Outgroups are *Prionodon linsang *(Pli) and *Cryptoprocta ferox *(Cfe) in the host tree, and *Procyon lotor *PV type 1 (PlPV1) and canine oral PV (COPV) in the virus tree. **(b) **Branch length correlation (nucleotide substitutions per site) for the feline and PV phylogenetic trees. Outgroup lineages were excluded from this analysis. FdPV1, *Felis domesticus *PV type 1; LrPV1, *Lynx rufus *PV type 1; PcPV1, *Puma concolor *PV type 1; PlpPV1, *Panthera leo persica *PV type 1; UuPV1, *Uncia uncia *PV type 1.

Phylogenetic trees of the feline PVs and of their feline host species were constructed using both maximum likelihood (ML) and Bayesian methods. The feline multigene tree (Figure 2a, left side), inferred from a Felidae total DNA alignment including both nuclear and mitochondrial DNA sequences [12], revealed identical tree topologies when ML and Bayesian methods were used for phylogenetic reconstruction. The closely related banded linsang (*Prionodon linsang*) and fossa (*Cryptoprocta ferox*), both of which are members of the Feliformia suborder of carnivores, but not members of the Felidae family, were used as outgroups for the Felidae phylogenetic tree. The feline PV phylogenetic tree (Figure 2a, right side), based on amino acid translation of the concatenated PV genes, also exhibited identical topologies when reconstructed with ML and Bayesian methods. Outgroups used in this analysis were the closely related PlPV1 of the raccoon and COPV of the dog, both belonging to the genus λ-PV and isolated from hosts belonging to the Caniformia suborder of carnivores. Our phylogenetic analysis revealed that the evolutionary history of feline PVs is identical to that established for the Felidae, suggesting that these PVs are indeed co-evolving with their hosts.

Similarities between virus and host phylogenies could, however, also arise through preferential host switching, in which viruses are transmitted more successfully between closely related hosts in geographic proximity, as is - for instance - the case for simian immunodeficiency virus [13]. The felid host species used in our study, however, are distributed over five distinct zoogeographic regions: Palearctic, Ethiopian (African), Nearctic, and Neotropical (including Southern Florida) [14]. This is demonstrated by the color code of the scientific names and branches in our host species phylogenetic tree (Figure 2a), which depicts recent and historic zoogeographic regions as shown on the map, inferred from their current distribution, fossil records, and phylogenetic analyses conducted by Johnson and coworkers [12]. In the feline PV evolutionary tree, the color of the virus name indicates the geographic region (shown on the map) where the virus was sampled. For all viruses, the geographic region where the virus was isolated coincides with the current distribution region of the corresponding feline species in the host tree, which is depicted by colored bars connecting virus and host. The only exception is PlpPV1, which was retrieved from a sample of an Asian lion (*Panthera leo persica *subspecies of *Panthera leo*) from the Gir Forest Sanctuary in India in the Oriental region, whereas the corresponding host sequence was obtained from a *Panthera leo *of the Ethiopian region (subspecies not defined [12]). Nevertheless, our findings indicate that the species-specific virus-host associations have remained stable throughout the intercontinental migration history of the felid lineage, making it unlikely that observed similarities between the felid and feline PV phylogenies are the result of preferential host switching. When the branch lengths of the Felidae and PV trees were compared, we found a strong linear relationship (*P *= 0.012; Figure 2b). This indicates that the accumulation of genetic diversity has occurred over similar amounts of time in both virus and host, and provides the necessary additional temporal support for virus-host co-evolution.

### Evolutionary rate estimation

**Figure 3:**
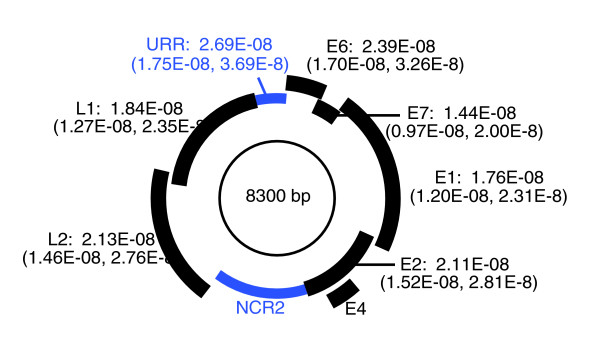
Feline papillomavirus evolutionary rates. Schematic representation of the circular feline papillomavirus (PV) genome with mean evolutionary rates (in nucleotide substitutions per site per year) and confidence intervals for the separate coding genes and the upstream regulatory region (URR). The URR and the second noncoding region (NCR2), which is unique for the PVs of the genus λ-PV, are shown in blue.

The fact that host and virus evolutionary tree branch lengths exhibit a strong linear correlation justifies applying host fossil calibrations to estimate viral evolutionary rates. We used a Bayesian statistical inference procedure that uses a relaxed molecular clock model to estimate evolutionary rates [15,16]. We incorporated dates and confidence intervals for each node in the host phylogeny, estimated using 16 independent fossil calibration points and 18 kilobases (kb) of sequence data [12], to constrain divergence dates within the viral phylogeny. This Bayesian MCMC inference resulted in a relatively precise estimate of the evolutionary rate of the feline PVs: 1.95 × 10^-8 ^(95% confidence interval 1.32 × 10^-8 ^to 2.47 × 10^-8^) nucleotide substitutions per site per year for the viral coding genome. Evolutionary rates for the individual PV coding genes and for the URR are indicated in Figure 3.

## Discussion

### Monophyletic origin of the Lambdapapillomaviruses

Based on the percentage nucleotide identity across the L1 ORF (provided in Additional data file 2), the novel feline PVs described here are classified in species 1 of the λ-PV genus, together with the domestic cat FdPV1. This genus also contains the dog COPV (in species 1) and the raccoon PlPV1 (in species 3). Apart from their high nucleotide similarity, additional support for a common ancestral origin of the PV types of this genus is provided by the presence of an unusual second noncoding region (NCR2). A multiple nucleotide sequence alignment of the NCR2 of the feline PVs demonstrated that these exhibit a high degree of similarity, with stretches of highly conserved nucleotides interrupted by variable regions (alignment is provided in Additional data file 3). The remarkable conservation of some stretches of nucleotides within the NCR2 of the feline PVs suggests that these might be of structural or functional importance. However, we were unable to detect any regulatory or structurally functional domains in these conserved regions. When we aligned the NCR2 regions of all λ-PVs using the T-coffee software [17], a moderate degree of similarity was still visible above the background of numerous insertions and deletions.

We therefore speculate that the NCR2 regions of all members of the λ-PV genus share a common evolutionary origin, having arisen in their common ancestor either through an early integration event with a DNA sequence of unknown function and origin or through duplication of a part of the PV genome, with subsequent loss of its superfluous function. (However, the latter possibility is not corroborated by detectable similarity of the NCR2 to any other region of the PV genome.) The NCR2 regions subsequently diverged in the descendent papillomaviral lineages through relatively rapid (compared with the coding genome) accumulation of insertions, deletions, and point mutations. The relatively high degree of conservation of the NCR2 sequence within the feline PVs, in contrast to the low similarity of the feline PV NCR2s to those of COPV and PlPV1, is a strong indication that the points of divergence between the various feline PVs occurred much more recently than that between the feline PVs and the PVs of the Caniformia. This is in agreement with a mode of evolution in which virus and host are co-diverging.

### Co-evolutionary relationships of feline papillomaviruses and their host species

Because of their rapid evolutionary rate relative to the available range of sequence sampling times, genetic diversity in rapidly evolving RNA viruses can be used as a genetic tag of their hosts, as was recently demonstrated for the feline immunodeficiency virus and its *Puma concolor *host [2]. With an evolutionary rate in the range of 10^-3 ^to 10^-5 ^nucleotide substitutions per site per year, these fast evolving pathogens are well suited for observing spatial and temporal evolutionary patterns on a 'real time' scale, and they can reflect short-term patterns of host movements (covering a few hundred years) [18,19]. In contrast, investigation of host evolution and demographic processes over time frames of hundreds of thousands to millions of years could be addressed by genetic variation in more slowly evolving viruses, which mainly (but not exclusively) include viruses with a stable DNA genome. Viruses for which the route of transmission is well established and requires very close direct contact, such as skin-skin and muco-mucosal transmission, provide excellent candidates for this approach. In order to infer information on host evolution from virus data with confidence, however, it is a prerequisite that the virus conforms to a vicariance model of evolution, with co-divergence of virus and host; which requires confirmation by rigorous testing.

The high similarities between the feline PV genomes described in this paper (Additional data file 1) strongly indicate that these feline PVs belong to the same ancestral PV lineage. The topology of the feline PV phylogenetic tree is identical to that of the host species tree, despite the complex and dynamic phylogeographic history of the Felidae. In addition, the strong linear relationship between the branch lengths for the Felidae and feline PV trees indicates that virus and host have accumulated genetic diversity over similar amounts of time, thereby fulfilling the temporal requirement for putative co-divergence. Although alternative hypotheses such as host switching and rapid adaptation to a new host possibly could also generate species-specific lineages and result in the percentage nucleotide identity among feline PVs that we found here, we argue that this was not the case for these feline PVs. First, the viral sequences were sampled from host species that are geographically isolated from each other, making interspecies jumping of these pathogens highly unlikely. Second, host switching followed by rapid adaptation would not result in an identical internal branching pattern for the viral and host evolutionary trees, such as we observed for the feline PVs and their hosts. Furthermore, the low overall nonsynonymous/synonymous substitution rate ratios (ω = dN/dS) observed in PVs, with comparison of complete genomes of HPV16 variants showing a ω below 1 for all ORFs (ranging from 0.1330 to 0.7966, depending on the ORF), indicates that PVs are under strong purifying selection pressure [20]. It would therefore be very speculative to hypothesize that PVs are capable of rapid adaptation to a new host.

The observed co-evolutionary pattern of feline PVs and their hosts provides evidence that slowly evolving viruses such as PVs can be used to investigate evolutionary processes in divergent host lineages. In combination with other data, they could help to unravel long-term evolutionary processes such as speciation and population subdivision. However, these findings can not be expanded to other slowly evolving viruses without prior confirmation of host co-divergence of the virus in question. This necessity is illustrated by phylogenetic studies of the human polyomavirus JC (JCV). This virus has widely been used as a genetic marker for human evolution and migration, based on presumed co-evolution with its human host. The hypothesis of virus-host co-divergence was based on the simple observation that subtypes of JCV are associated with different human populations, and that the virus is transmitted from parent to child in a quasi-vertical manner. However, a recent reconciliation analysis of human and JCV phylogenetic trees was unable to provide evidence for co-divergence between virus and host, indicating that this virus is unsuited as a marker to unravel the history of human populations [19].

### Feline papillomavirus evolutionary rate

The observed co-evolutionary pattern between feline PVs and their hosts justifies applying host species divergence times to estimate viral evolutionary rates. Our results show that substitutions accumulate most rapidly in the noncoding upstream regulatory region (with 2.69 × 10^-8 ^nucleotide substitutions per site per year; Figure 3). For the viral coding genes, we found the highest evolutionary rate in the E6 ORF and the lowest in E7. We wish to emphasize that, taking into account the confidence intervals, the evolutionary rates for the separate coding genes largely overlap. In a previously report by Bravo and Alonso [21] on divergence rates in mucosal HPVs, the protein divergence rate was reported to increase in the order L1 < L2 < E6 ≈ E7. However, because of a lack of calibration points, no time scale was attributed to the HPV phylogeny in that study, and so calculations were limited to relative measurements based on divergence distances.

The overall evolutionary rate of 1.95 × 10^-8 ^nucleotide substitutions per site per year for the coding genome of the feline PVs that we estimated here is in broad agreement with previous point estimates for rates of PV evolution based on simple genetic distances, few calibration points, and a limited number of genes [8]. Furthermore, our estimate is within the same range as the HPV evolutionary rate, which was approximated by Ong and colleagues [22] based on comparison of HPV18 variants from isolated human populations. They established that the fixation of a single point mutation in a 185 bp fragment of the noncoding region (URR) takes at least 12,000 years, resulting in a rough estimate of evolutionary rate of maximally 4.5 × 10^-7 ^nucleotide substitutions per site per year for the most variable region of the HPV18 genome. It is important to note that this estimate is based on the assumption of co-evolution of HPV18 with human populations, which was not validated by rigorous testing in that study. This rate approximation is only about five times faster than our precise estimates of the URR evolutionary rate (2.69 × 10^-8 ^nucleotide substitutions per site per year, with a 95% confidence interval of 1.75 × 10^-8 ^to 3.69 × 10^-8^).

Our calculations indicate that mutations are fixed in the PV genome at a very low rate. This is primarily the result of a low rate of occurrence of mutations, because PVs use the host cell DNA replication machinery, characterized by high fidelity, proofreading capacity, and post-replication repair mechanisms. Furthermore, a negative selective pressure acting on the PV genome, indicated by a nonsynonymous/synonymous substitution rate ratio below 1 for all ORFs [20], will prevent the fixation of deleterious mutations in the PV population, thereby contributing to the low rate of evolution. Although the papillomaviral DNA is replicated by the host cell machinery, our rate estimates indicate that PV genes evolve approximately one order of magnitude faster than mammalian host cellular genes, which have a mutation rate of less then 1 × 10^-9 ^nucleotide substitutions per site per year [23]. A greater number of replication cycles per unit time, and thus faster generation times, provides a possible explanation for these higher viral rates.

Well supported evolutionary rates of the feline PVs could possibly be applied to other PV lineages and associated evolutionary questions. For example, diversity within the sexually transmitted HPV types 16 and 18, which worldwide are the major causes of cervical cancer, has been suggested to mirror past human migrations [22,24,25]. However, until now, rate estimates for PV evolution had not been sufficiently precise to estimate a reliable time scale for this migration history [1]. By applying the evolutionary rate that we have now calculated, the time to most recent common ancestor of the extant variants of HPV16 and HPV18, and the demographic history of the viral population over time could be estimated and compared with the host data. Given the very low degree of diversity among these HPV variants, the current dataset, encompassing only a very limited number of complete genomic sequences of HPV16 and HPV18 variants, will probably be insufficient to obtain precise estimates. As more sequence data become available in the future, however, these studies could result in an accurate time scale for HPV evolution, and possibly provide pivotal information about global patterns of population dispersal and interaction in human evolutionary history, where traditional genetic studies have fallen short.

### Papillomavirus infection in endangered species

Nearly all Felidae, except for the domestic cat, are presently listed as endangered species by the World Wildlife Fund and are monitored for conservation concerns. An increased prevalence of PV infection and more severe PV-related disease has been reported in populations that experienced a genetic bottleneck in the past. For instance, oral focal epithelial hyperplasia (FEH), caused by HPV13 and HPV32, is highly prevalent in American Indians in the USA and Brazil, and in Inuits in Greenland and Alaska, but it is only rarely encountered in non-inbred populations [26]. In the endangered primate species *Pan paniscus *(pygmy chimpanzee or bonobo), the pygmy chimpanzee PV causes very similar oral lesions, also referred to as FEH [27]. Interestingly, oral lesions with the clinical and histopathologic features of FEH have also been described in the bobcat, Florida panther, Asian lion, and snow leopard, in which they could be caused by LrPV1, PcPV1, PlpPV1, and UuPV1, respectively [6]. A major concern is that PV infections in endangered exotic felids could pose important health problems and even jeopardize the survival of these species. Transformation of papillomatous lesions to cutaneous squamous cell carcinoma, with an effective mortality rate of 100%, has been reported in captive snow leopards, and could compromise the zoo population of these animals [28]. The construction of PV type-specific prophylactic (or even therapeutic) vaccines could therefore be considered, as has been done previously for cattle, dogs, rabbits, and horses [29-31]. The sequence information of the LrPV1, PcPV1, PlpPV1, and UuPV1 genomes, provided in this report, is essential to the development of such vaccines.

## Conclusion

Recently, molecular data from the rapidly evolving feline immunodeficiency virus contributed to resolving the population history of its *Puma concolor *host [2]. Although this clearly shows that fast evolving viruses are ideal for reconstructing the demographic history of a single host species, we have now tested the hypothesis that more slowly evolving viruses may be useful for addressing relationships on a broader evolutionary scale. To this effect, we have sequenced the complete genomes of several globally distributed feline PVs. A confident reconstruction of the feline host phylogeny recently became available [12], enabling rigorous comparison of viral and host phylogenies. We demonstrate that the evolutionary relationships among feline PVs perfectly mirror those of their hosts, despite a complex and dynamic phylogeographic history. By applying host species divergence times and using Bayesian statistical methods and a relaxed molecular clock, we have calculated the first precise estimates for the rate of evolution for each of the PV genes. Our results provide evidence for long-term virus-host co-speciation of these feline PVs, indicating that viral diversity in slowly evolving viruses can be used to investigate host species evolution. Our evolutionary rate estimates provide the cornerstones for further studies on the ancient pandemic spread of these viruses in a plethora of different species.

## Materials and methods

### Papillomavirus samples

Biopsy material was obtained from oral papillomas from a bobcat and a Florida panther, both of which were wild, free-living individuals in the Big Cypress Swamp of southern Florida; from a free-living Asian lion from the Gir Forest Sanctuary in India; and from a snow leopard from the Seattle Woodland Park Zoo. From this biopsy material, total genomic DNA was isolated by phenol-chloroform-isoamylalcohol extraction, as described previously [32].

### Multiply primed rolling-circle amplification and cloning

Multiply primed rolling-circle amplification (RCA) was performed on extracted DNA of the papillomatous lesions of the bobcat, Florida panther, and Asian lion, by using the TempliPhi 100 Amplification kit (Amersham Biosciences, Roosendaal, The Netherlands) following a recently optimized protocol for amplification of PV complete genomic DNA [32]. One microliter of extracted DNA (containing 2.71 μg, 0.35 μg, and 1.98 μg of total DNA for the bobcat, Florida panther, and Asian lion, respectively) or water (negative control) as input material was used for RCA, and reactions were carried out as described previously [33]. To investigate whether PV DNA was amplified, 2 μl of the RCA products was digested with 10 U of *BamH*I, *EcoR*I, *Hind*III, *Sal*I, and *Xba*I. After digestion, the products were run on a 0.8% agarose gel to check for the presence of a DNA band consistent with full-length PV DNA (about 8 kb), or multiple bands with sizes adding up to this length. DNA fragments of PV complete genomic size were found after digestion of the bobcat, Florida panther, and Asian lion RCA-amplified DNA with *Xba*I, *Sal*I, and *EcoR*I, respectively. For cloning of these fragments, 10 μl of RCA product was digested with 100 U of the respective restriction enzyme, and run on a 0.8% agarose gel, after which the 8 kb fragments were cut out from the gel, and DNA was extracted from the gel slices using GeneClean (BIO 101 Systems/Qbiogene, Carlsbad, CA, USA). The DNA fragments were ligated into de-phosphorylated *Xba*I-, *Sal*I-, or *EcoR*I-cut pUC18 respectively. After transformation of One Shot MAX Efficiency DH5α-T1R competent cells (Invitrogen, Merelbeke, Belgium), the bacteria were incubated for blue-white colony screening on agar plates containing X-gal, and white colonies were checked by restriction digestion of miniprep DNA. One clone containing the 8 kb fragment was selected for each of the PV genomes.

### Degenerate primer PCR, long template PCR, and cloning

Polymerase chain reaction (PCR) reactions with degenerate PV-specific primer pairs AR-L1F1/AR-L1R3, AR-L1F1/AR-L1R5, and AR-E1F2/AR-E1R3 were performed on the snow leopard extracted DNA as described previously [33]. Amplicons with a size consistent with PV-specific amplification were purified through 3% agarose gel electrophoresis and extraction of the PV-specific bands (QIAquick Gel Extraction Kit; QIAgen, Venlo, The Netherlands), and were sequenced with the same degenerate primers as used for PCR. Primers for long template PCR were chosen in the partial E1 and L1 sequences obtained by degenerate primer PCR on the snow leopard sample. Two overlapping long PCR fragments, together encompassing the entire PV genome, were amplified by long template PCR. Primer pairs used were UuPV-F1 in L1 (5'-CATGCAAGCAGCGATCGCCTCTTGACAGTCGG-3') and UuPV-R1 in E1 (5'-ATTTGACTACTTCCTTCCAGTCTCCGTCGCC-3') for amplification of a fragment of approximately 3.7 kb (UuPV-F1R1), and UuPV-F2 in E1 (5'-AACTCTTGCTGACACAGATGAGAATGCAGCAGC-3') and UuPV-R2 in L1 (5'-ACTGTTGGCTCTCGTGGCCTGCCCCATTACACC-3') for amplification of a second fragment of approximately 5.3 kb (UuPV-F2R2).

Long template PCR was carried out using the Expand Long Template PCR System (Roche Diagnostics, Vilvoorde, Belgium), in accordance with the manufacturer's instructions. The amplification profile consisted of 2 min denaturation at 94°C, followed by 10 cycles of 10 s denaturation at 94°C, 30 s annealing at 65°C, and 4 min elongation at 68°C, and in the following 20 cycles the elongation time was extended with 20 s for each cycle. After a final elongation step of 7 min at 68°C, the samples were cooled to 4°C. The long template PCR products were checked by agarose gel electrophoresis and ethidium bromide staining. The UuPV-F1R1 and UuPV-F2R2 fragments were gel purified and cloned into pCR-XL-TOPO vector, followed by transformation into One Shot TOP 10 chemically competent cells (Invitrogen). The bacteria were selectively grown on LB agar plates containing 50 μg/ml kanamycin. For each PCR fragment, 10 colonies were checked by *EcoR*I digest of miniprep DNA, and one clone containing UuPV-F1R1 and one containing UuPV-F2R2 were selected.

### DNA sequencing

The complete nucleotide sequences of the cloned bobcat LrPV1, the Florida panther PcPV1, and the Asian lion PlpPV1 were determined by using the EZ::TN < KAN-2 > Insertion Kit, whereas for the snow leopard UuPV1 the EZ::TN < TET-1 > Insertion Kit was used (Epicentre, Madison, WI, USA). These methods allow rapid sequencing of long stretches of cloned DNA by random insertion of a transposon containing specific forward and reverse sequencing primer binding sites. Transposon insertion reactions were performed on miniprep DNA of the LrPV1, PcPV1, PlpPV1, UuPV-F1R1, and UuPV-F2R2 selected clones, after which the reaction products were used to transform One Shot MAX Efficiency DH5α-T1R competent cells (Invitrogen). Sequencing with forward and reverse primers (the KAN-2 FP-1 and KAN-2 RP-1, and the TET-1 FP-1 and TET-1 RP1, respectively; Epicentre) was performed on 24 clones obtained from each of the transposon insertion reactions, and the remaining sequence gaps were covered by primer-walking on the original clones. Sequencing was performed on an ABI Prism 3100 Genetic Analyzer (Perkin-Elmer Applied Biosystems, Foster City, CA, USA) at the Rega Institute core sequencing facility. Chromatogram sequencing files were inspected with Chromas 2.2 (Technelysium, Helensvale, Australia), and contigs were prepared using SeqMan II (DNASTAR, Madison, WI, USA).

### Sequence analysis

ORF analysis was performed using the ORF Finder tool on the NCBI server. Similarity searches were performed using the NCBI Basic Local Alignment Search Tool (BLASTN 2.2.10) server on GenBank DNA database release 145.0 [34]. The predicted molecular weight of the putative proteins was calculated using the ExPASy (Expert Protein Analysis System) Compute pI/Mw tool [35]. Pair-wise sequence alignments were calculated using the GAP-program [36] (percentage similarity is provided in Additional data file 2). For aligning sequences, different programs were applied to the data in order to check the robustness of the alignment with respect to the different algorithms that were applied. The program giving the best alignment was then selected, and this alignment was taken as the starting point for manual correction. The URR alignment of the λ-PVs (Figure 1), and the NCR2 alignment of the feline PVs (Additional data file 3) were obtained using ClustalX version 1.83 [37] and T-Coffee [17], and corrected manually in the GeneDoc Multiple Sequence Alignment Editor and Shading Utility software package version 2.6.002 [38].

### Phylogenetic analysis

PV types included for phylogenetic analysis were the feline PVs FdPV1 (GenBank: NC_004765), LrPV1 (AY904722), PcPV1 (AY904723), PlpPV1 (AY904724), and UuPV1 (DQ180494); the nonfeline λ-PVs COPV (NC_001619) and PlPV1 (AY763115) were used as outgroup sequences. Sequences of the various separate PV genes were aligned using the program Muscle [39]. Alignments of the E6, E7, E1, E2, L2, and L1 genes were combined into one concatenated alignment (Additional data file 4). Felidae nuclear and mitochondrial DNA sequences of the domestic cat (*Felis catus*), puma (*Puma concolor*), bobcat (*Lynx rufus*), lion (*Panthera leo*), and snow leopard (*Uncia uncia *or *Panthera uncia*), with the banded linsang (*Prionodon linsang*) and the fossa (*Cryptoprocta ferox*) as nonfeline outgroups, were obtained from Johnson and coworkers [12]. We retrieved the nucleotide alignment from Table S7 in the supporting online information provided by Johnson and coworkers [40], and we deleted sites that could not be unambiguously aligned according to the original authors. The alignment for the cat, puma, bobcat, lion, snow leopard, linsang, and fossa sequences was 22,635 bp long in total and contains autosomal, X-linked, Y-linked, and mitochondrial genes (18,702 bp of nuclear DNA and 3,933 bp mitochondrial DNA). A detailed list of these genes, their felid chromosomal location, and the number of base pairs for each gene segment (excluding ambiguous sites) is provided in Table S1 of the supporting online information provided by Johnson and coworkers [40]. The mean pair-wise divergence for these sequences is estimated to be 0.0896802 (95% confidence interval 0.0861073 to 0.0933866) nucleotide substitutions per site.

Phylogenetic analyses of both virus and host alignments were performed using model-based statistical inference techniques, including maximum likelihood methods implemented in Tree-puzzle 5.1 [41] and Bayesian methods as implemented in MrBayes 3.1 [42]. Analyses were performed on the translated amino acid sequence data for PVs using the JTT (Jones, Taylor and Thornton) substitution model [43] and gamma-distributed rate variation in Tree-puzzle [44], and using gamma-distributed rate variation but estimating the fixed-rate model of substitution in MrBayes [42]. According to the Akaiki information criterion and the Bayesian estimation procedure, the JTT model was the best fitting amino acid substitution model. The Felidae total DNA (totDNA) alignment, including both nuclear and mitochondrial DNA, was analyzed using the Tamura-Nei model of substitution and gamma-distributed rate variation in Tree-puzzle [44] and the general-time-reversible model with gamma-distributed rate variation in MrBayes [42]. Bayesian MCMC analyses in MrBayes [42] were run incorporating flat priors, random starting trees, and no phylogenetic constraints. Four simultaneous Markov chains were run for 5,000,000 generations, with 500,000 generations of burn-in followed by sampling every 5,000 generations. Each Bayesian run was independently replicated to ensure convergence. Branch lengths for both virus and host nucleotide alignments were estimated using the general-time-reversible model with gamma-distributed rate variation in PAUP* 4b10 [45].

### Evolutionary rate estimation

Evolutionary rates were estimated using an uncorrelated relaxed clock in BEAST 1.3 [15]. Bayesian MCMC analyses in BEAST were performed using the Hasegawa-Kishino-Yano model of evolution with gene-specific gamma-distributed rate heterogeneity among sites and gene-specific evolutionary rates; rates on each branch were drawn identically and independently from an underlying log-normal distribution. Monophyletic constraints were imposed for the nodes that were used to calibrate the evolutionary rates. Normal priors were used for the times to the most recent common ancestor (TMRCA) of PcPV1, LrPV1, FdPV1, PlpPV1, and UuPV1 (mean 10.78 million years ago (Mya); standard deviation 1.87), for the TMRCA of PcPV1, LrPV1 and FdPV1 (mean: 7.15 Mya, stdev:1.36) and for the TMRCA of PlpPV1 and UuPV1 (mean: 3.72 Mya, stdev:1.05), all based on the posterior distributions obtained for the host [12]. The divergence dates reported by Johnson and coworkers were estimated using a Bayesian relaxed clock approach that models the molecular rate among lineages as varying in an autocorrelated manner. The phylogeny used to estimate divergence times by those authors was a maximum likelihood tree inferred from the amino acid sequences for the nuclear genes (Figure S1 in their supporting online information [40]). The 16 calibration dates that were used to infer the time scale for the host phylogeny are described in detail under the paragraph 'Divergence time estimations' in that supporting online information.

We have run MCMC chains in BEAST for 5,000,000 generations, with burn-in and sampling as described above [15]. The URR evolutionary rate was estimated separately on an alignment of the URR sequences of the feline PVs without outgroup sequences, constructed in Muscle (the URR alignment is available in Additional data file 5).

### Papillomavirus nomenclature

The novel PVs described in this study were named and abbreviated according to the nomenclature guidelines for nonhuman PVs (abbreviation 'PV', preceded by a capital and a lower case letter that abbreviate the host's scientific name) [9]: LrPV1, PcPV1, and UuPV1. An exception was made for the *Panthera leo persica *PV type 1, which was abbreviated PlpPV1 instead of PlPV1, since the latter name is already in use for the *Procyon lotor *PV type 1, isolated from a raccoon [11].

### Nucleotide sequence accession numbers

The complete genomic sequences of the four novel feline PVs were deposited in GenBank under accession numbers (GenBank: AY904722, AY90472, AY904724, DQ180494) for LrPV1, PcPV1, PlpPV1, and UuPV1, respectively.

## Additional data files

The following additional data are available with the online version of this paper. Additional data file 1 specifies the positions of the various ORFs of LrPV1, PcPV1, PlpPV1, UuPV1, and FdPV1. Additional data file 2 shows the percentages nucleotide and amino acid similarity of the various ORFs of the feline PVs mutually and to the corresponding ORFs of COPV, HPV1, HPV5, HPV16, and BPV1. Additional data file 3 contains the NCR2 alignment of the feline PVs. Additional data file 4 contains the concatenated alignment of the different coding genes of the members of the λ-PV genus. Additional file 1 contains the alignment of the URR sequences of the feline PVs.

## Supplementary Material

Additional data file 1 Table indicating the nucleotide position of the start of the ORF, start codon, and stop codon, the nucleotide and amino acid count of the ORFs and the predicted proteins respectively, and the predicted molecular weight of the putative proteins, for the different ORFs of the feline PVs LrPV1, PcPV1, PlpPV1, UuPV1, and FdPV1. Click here for file

Additional data file 2 Table indicating the percentages nucleotide and amino acid similarity of different ORFs of the feline PVs mutually and with the corresponding ORFs of COPV, HPV1, HPV5, HPV16, and BPV1. Click here for file

Additional data file 3 Nucleotide sequence alignment of the NCR2 of the feline PVs LrPV1, PcPV1, PlpPV1, UuPV1, and FdPV1, from the stop codon of E2 to the start codon of L2. Nucleotides are shaded according to the degree of conservation (black = 100% conserved; dark gray = 80% to 99% conserved; light gray = 60% to 79% conserved; no shade = <60% conserved). Click here for file

Additional data file 4 Concatenated alignment of the E6, E7, E1, E2, L2, and L1 ORFs of the members of the genus λ-PV, used for phylogenetic reconstruction and calculation of evolutionary rates. Click here for file

Additional data file 5 Alignment of the URR (NCR1) of the feline PVs, used for calculation of the URR evolutionary rate. Click here for file
