# Supplementary material for: Ancient papillomavirus-host co-speciation in Felidae
Source: Genome Biol. 2007 Apr 12;8(4):R57. doi: 10.1186/gb-2007-8-4-r57 (PMC1896010; doi:10.1186/gb-2007-8-4-r57)
Supplement: Additional data file 1 — Table indicating the nucleotide position of the start of the ORF, start codon, and stop codon, the nucleotide and amino acid count of the ORFs and the predicted proteins respectively, and the predicted molecular weight of the putative proteins, for the different ORFs of the feline PVs LrPV1, PcPV1, PlpPV1, UuPV1, and FdPV1. [file gb-2007-8-4-r57-S1.doc]

**Table 1. Position of open reading frames of LrPV1, PcPV1, PlpPV1, UuPV1 and FdPV1.**

| **ORF** | | **Nucleotide position** | | | **# nt^1^** | **# AA^2^** | **MW (kDa)^3^** |
| --- | --- | --- | --- | --- | --- | --- | --- |
|  |  | **Start ORF** | **Start codon** | **Stop codon** |  |  |  |
| E6 | LrPV1 | 1 | 43 | 457 | 456 | 138 | 15.4 |
|  | PcPV1 | 1 | 31 | 445 | 444 | 138 | 15.6 |
|  | PlpPV1 | 1 | 115 | 529 | 548 | 138 | 15.5 |
|  | UuPV1 | 1 | 31 | 445 | 444 | 138 | 15.3 |
|  | FdPV1 | 111 | 141 | 555 | 444 | 138 | 15.2 |
| E7 | LrPV1 | 420 | 456 | 738 | 318 | 94 | 10.2 |
|  | PcPV1 | 408 | 444 | 732 | 324 | 96 | 10.5 |
|  | PlpPV1 | 504 | 528 | 813 | 309 | 95 | 10.4 |
|  | UuPV1 | 408 | 444 | 726 | 318 | 94 | 10.2 |
|  | FdPV1 | 518 | 554 | 839 | 321 | 95 | 10.4 |
| E1 | LrPV1 | 727 | 730 | 2554 | 1827 | 608 | 69.4 |
|  | PcPV1 | 697 | 724 | 2542 | 1845 | 606 | 687 |
|  | PlpPV1 | 787 | 805 | 2623 | 1836 | 606 | 69.0 |
|  | UuPV1 | 700 | 718 | 2536 | 1836 | 606 | 68.9 |
|  | FdPV1 | 813 | 831 | 2652 | 1839 | 607 | 68.8 |
| E2 | LrPV1 | 2474 | 2498 | 3638 | 1164 | 380 | 43.5 |
|  | PcPV1 | 2462 | 2486 | 3611 | 1149 | 375 | 42.3 |
|  | PlpPV1 | 2543 | 2567 | 3707 | 1164 | 380 | 43.0 |
|  | UuPV1 | 2556 | 2480 | 3620 | 1164 | 380 | 42.7 |
|  | FdPV1 | 2572 | 2596 | 3739 | 1167 | 381 | 43.0 |
| E4 | LrPV1 | 3075 | / | 3402 | 327 | 109 | 12.5 |
|  | PcPV1 | 3063 | / | 3375 | 312 | 104 | 12.0 |
|  | PlpPV1 | 3144 | / | 3471 | 327 | 109 | 12.5 |
|  | UuPV1 | 3057 | / | 3384 | 327 | 109 | 12.1 |
|  | FdPV1 | 3170 | / | 3503 | 333 | 111 | 12.7 |
| L2 | LrPV1 | 4811 | 4844 | 6371 | 1560 | 509 | 54.8 |
|  | PcPV1 | 4866 | 4899 | 6444 | 1578 | 515 | 55.5 |
|  | PlpPV1 | 4737 | 4770 | 6303 | 1566 | 511 | 55.3 |
|  | UuPV1 | 4629 | 4662 | 6198 | 1569 | 512 | 55.2 |
|  | FdPV1 | 4980 | 5013 | 6540 | 1560 | 509 | 54.9 |
| L1 | LrPV1 | 6316 | 6385 | 7882 | 1566 | 499 | 56.9 |
|  | PcPV1 | 6344 | 6458 | 7958 | 1614 | 500 | 56.9 |
|  | PlpPV1 | 6306 | 6318 | 7824 | 1518 | 502 | 57.0 |
|  | UuPV1 | 6169 | 6214 | 7714 | 1545 | 500 | 56.9 |
|  | FdPV1 | 6407 | 6467 | 8054 | 1647 | 529 | 60.3 |

^1^ Number of bases from the first base of the ORF until the last base of the last codon before the stop codon.

^2^ Number of amino acids that would produce the predicted proteins if translation starts at the first ATG of the ORF, except for E4 which does not possess a start codon (indicated by /) and therefore hypothetically begins at the first in frame amino acid.

^3^ Predicted molecular weight of the putative protein, calculated using the ExPASy (Expert Protein Analysis System) Compute pI/Mw tool.
