# Supplementary material for: Ancient papillomavirus-host co-speciation in Felidae
Source: Genome Biol. 2007 Apr 12;8(4):R57. doi: 10.1186/gb-2007-8-4-r57 (PMC1896010; doi:10.1186/gb-2007-8-4-r57)
Supplement: Additional data file 2 — Table indicating the percentages nucleotide and amino acid similarity of different ORFs of the feline PVs mutually and with the corresponding ORFs of COPV, HPV1, HPV5, HPV16, and BPV1. [file gb-2007-8-4-r57-S2.doc]

**Table 2. Percentages similarity*** of LrPV1, PlpPV1, PcPV1 and UuPV1 ORFs mutually and to other PVs.

| **ORF** | | **LrPV1** | **PcPV1** | **PlpPV1** | **FdPV1** | **COPV** | **HPV1** | **HPV5** | **HPV16** | **BPV1** |
| --- | --- | --- | --- | --- | --- | --- | --- | --- | --- | --- |
| E6 | LrPV1 |  |  |  | 78 (75) | 45 (36) | 44 (31) | 38 (29) | 35 (20) | 28 (23) |
|  | PcPV1 | 80 (72) |  |  | 76 (72) | 44 (39) | 47 (36) | 37 (29) | 33 (20) | 34 (26) |
|  | PlpPV1 | 71 (65) | 74 (65) |  | 74 (68) | 44 (36) | 42 (34) | 40 (29) | 34 (20) | 35 (23) |
|  | UuPV1 | 72 (63) | 72 (63) | 80 (76) | 73 (65) | 44 (35) | 48 (36) | 39 (27) | 32 (20) | 36 (23) |
| E7 | LrPV1 |  |  |  | 87 (83) | 56 (53) | 43 (31) | 44 (34) | 34 (31) | 26 (17) |
|  | PcPV1 | 86 (82) |  |  | 87 (84) | 58 (55) | 44 (33) | 47 (32) | 46 (32) | 25 (17) |
|  | PlpPV1 | 82 (80) | 86 (82) |  | 83 (81) | 57 (52) | 46 (36) | 43 (35) | 41 (31) | 23 (18) |
|  | UuPV1 | 83 (79) | 86 (80) | 88 (84) | 85 (84) | 56 (55) | 42 (31) | 39 (32) | 33 (30) | NA (20) |
| E1 | LrPV1 |  |  |  | 79 (82) | 65 (61) | 57 (52) | 56 (46) | 49 (43) | 50 (40) |
|  | PcPV1 | 82 (85) |  |  | 78 (81) | 66 (61) | 56 (53) | 57 (48) | 44 (45) | 50 (42) |
|  | PlpPV1 | 80 (83) | 80 (81) |  | 79 (80) | 66 (60) | 58 (51) | 57 (48) | 49 (43) | 50 (40) |
|  | UuPV1 | 81 (83) | 80 (82) | 88 (91) | 79 (80) | 66 (61) | 57 (51) | 56 (47) | 48 (43) | 51 (41) |
| E2 | LrPV1 |  |  |  | 71 (66) | 56 (48) | 47 (40) | 36 (29) | 38 (33) | 34 (25) |
|  | PcPV1 | 77 (69) |  |  | 71 (65) | 58 (47) | 48 (39) | 36 (30) | 33 (32) | 34 (27) |
|  | PlpPV1 | 77 (73) | 74 (67) |  | 72 (67) | 56 (50) | 56 (39) | 37 (31) | 37 (31) | 33 (27) |
|  | UuPV1 | 75 (72) | 73 (67) | 85 (82) | 70 (66) | 57 (49) | 48 (39) | 37 (30) | 42 (32) | 34 (25) |
| E4 | LrPV1 |  |  |  | 66 (55) | 46 (36) | 40 (24) | 22 (12) | NA (NA) | 25 (NA) |
|  | PcPV1 | 71 (60) |  |  | 65 (51) | 52 (34) | 39 (25) | 15 (9) | NA (NA) | 26 (NA) |
|  | PlpPV1 | 74 (61) | 71 (57) |  | 69 (57) | 45 (31) | 35 (28) | 16 (11) | 28 (19) | 26 (NA) |
|  | UuPV1 | 70 (50) | 68 (53) | 77 (58) | 64 (47 | 43 (33) | 39 (22) | 20 (NA) | 27 (NA) | 31 (NA) |
| L2 | LrPV1 |  |  |  | 77 (82) | 59 (59) | 50 (43) | 46 (39) | 35 (30) | 31 (29) |
|  | PcPV1 | 79 (82) |  |  | 77 (82) | 59 (58) | 48 (42) | 45 (39) | 35 (30) | 35 (28) |
|  | PlpPV1 | 78 (83) | 77 (80) |  | 76 (83) | 62 (61) | 50 (38) | 45 (40) | 37 (30) | 35 (29) |
|  | UuPV1 | 76 (82) | 77 (81) | 84 (90) | 74 (81) | 60 (60) | 49 (41) | 47 (40) | 32 (30) | 33 (28) |
| L1 | LrPV1 |  |  |  | 77 (88) | 68 (74) | 57 (57) | 55 (52) | 52 (47) | 53 (47) |
|  | PcPV1 | 85 (94) |  |  | 77 (88) | 68 (74) | 58 (55) | 55 (52) | 53 (47) | 53 (48) |
|  | PlpPV1 | 79 (90) | 79 (89) |  | 73 (85) | 67 (74) | 58 (56) | 55 (53) | 52 (47) | 52 (47) |
|  | UuPV1 | 79 (89) | 78 (89) | 85 (93) | 73 (86) | 67 (74) | 59 (57) | 54 (52) | 51 (46) | 53 (48) |

* Percentages nucleotide (amino acid) identity, calculated by pairwise alignments.

NA: Not alignable because of insufficient similarity.
